# 

**Published:** 1884-07-20

**Authors:** 


### EDITORIALS AND MISCELLANEOUS.

### *READ! READ!!*

☞ Many of our subscribers are in arrears. Friends, the aggregate of these small amounts is very important to us. Please let us hear from you at ONCE.

R. C. WORD,
Managing Editor.

### *EDITORIAL NOTICES.*

MISSOURI MEDICAL COLLEGE.—See advertisement of this Institution, located at St. Louis, Mo.

LACTOPEPTINE holds its reputation as a useful aid to digestion. In the summer diarrhœas of children it has attained a high reputation. It may be given in connection with the food taken, and is said to prevent, in many cases, the indigestion which is a frequent cause of these bowel derangements. See advertisement in this Journal of New York Pharmacal Association.

WE have received a beautiful picture of the Southern Exposition, which opens at Louisville, Ky., August 16th, and continues until October 25th. The view is of the main building, which is one of the largest exposition buildings ever erected. It covers thirteen acres of ground, and will be lighted throughout by five thousand electric lights.

AMERICAN MEDICAL ASSOCIATION.—At the meeting of the American Medical Association held at Washington in May last, an amendment to Regulation II was adopted, which provides that

> Membership in the Association shall be obtainable by any member of a State or County Medical Society recognized by the Association, upon application endorsed by the President and Secretary of said Society; and shall be retained so long as he shall remain in good standing in his local Society, and shall pay his annual dues to the Association.

URINARY TEST PAPERS.—MESSRS. PARKE, DAVIS & CO., of Detroit, have placed the profession under renewed obligations in the preparation of neat little pocket cases, containing urinary test papers and apparatus for testing urine. The agents used are based upon suggestions in a paper published in The London Lancet, Feb. 3d, 1883, by Dr. G. Oliver; also upon a paper by Chas. W. Purdy, M.D., of Chicago, in the Journal of American Medical Association, January, '84. Full instructions are found in the case, which—from its convenience and the simplicity of the methods recommended—must prove very desirable and acceptable to practitioners.

VALERIAN.—Valerian has long been recognized by the profession as possessing valuable properties in a certain class of nervous affections. We are satisfied, from much experience in the use of this agent, that it is not sufficiently appreciated. It is a superior remedy in all hysterical and nervous affec tions, especially in those peculiar disorders connected with the menopause, or change of life, in females. It is our most efficient remedy in hysterical insomnia, in palpitation resulting from menstrual disorders, in nervous vertigo, in "dysphagia, borborygmi, flatulence, burning in the intestines, anxiety of the precordia, panic fears" and, in short, in nervous troubles generally.

It is unfortunate that, possessing such admirable qualities as a nervine, it has a most repulsive odor, a fact which renders it exceedingly obnoxious, especially to the particular class of patients who need it most. The fastidiousness of this class of patients is usually in proportion to their nervousness.

A preparation of the drug that would avoid its disagreeable odor, and yet retain the full powers of the medicine, has heretofore been a desideratum which was thought to be insurmountable; but we have now the satisfaction of announcing that the difficulties have been overcome by that enterprising and indomitable house, PARKE, DAVIS & Co., OF DETROIT.

These gentlemen have kindly sent us samples of the drug in three forms, skillfully and beautifully prepared, each of which we have found free from disagreeable odor, and efficient in action. The profession will, doubtless, welcome these new preparations, to-wit: Gelatine-coated Pills of the Abstract of Valerian, Gelatine-coated Pills of the Solid Extract of Valerian, and Elastic Capsules of the Oil of Valerian.

MEDICAL STUDENTS.—There is now no necessity for our students of the South to go North or to Europe to obtain a medical education. Ample facilities are to be found in certain of our home institutions for a thorough medical education. Let our young men spend their money at home, and thus aid in the building up of our home institutions. If, after securing your diploma at home, you desire to spend a winter abroad to compare methods and plans of teaching, and the better to impress upon the mind what has been learned, it is well to do so, and this may be done with comparatively little expense, as no charge is made, or, at least, very low rates are charged those who hold diplomas, and the graduated student is better prepared, and is more competent, to understand what he sees than one who, for the first time, enters upon a new and untried field of study.

### RECEIPTED.

1884.—Drs. H. T. Shiells, J. M. Jackson, W. J. Rogers, Powe'l & Sells. R. J. McMullin, S. L. Lockwood, T. A. Boggan, D. B. Searcy, D. R. Norman, J. E. Martin, F. L Fish, R. S. Roland, I. T. McGrath, S. P. Thomas, James McWilliams, Thos. A. Morton, J. B. Medlock to April, L. N. Hyten to July, R. D. Jackson to June, S. P. Clayton to July, M. Amen to April, A. D. Coleman to May.

1883.—G. L. Mills, B. F. Chambliss to July.

1885.—J. A. Williams.

